# Perceived blame risk and innovative behavior among Chinese grassroots civil servants: the roles of career calling and perceived organizational support

**DOI:** 10.3389/fpsyg.2026.1867276

**Published:** 2026-08-05

**Authors:** Huan Zhao, Xingyu Zhang, Peifeng Chen, Jinfeng Zhang, An Hu

**Affiliations:** 1 College of Computer Science, Chongqing University, Chongqing, China; 2 School of Public Policy and Administration, Chongqing University, Chongqing, China; 3 Chongqing Radio and Television Technology Center, Chongqing, China

**Keywords:** career calling, grassroots civil servants, innovative behavior, perceived blame risk, perceived organizational support

## Abstract

**Introduction:**

Grassroots civil servants operate at the forefront of public service, where they directly address complex social issues and exercise substantial discretion. As such, they represent a vital source of innovation in advancing the modernization of China’s governance system and capacity. However, in an accountability-driven administrative context, the fear of being blamed for potential failures may inhibit their willingness to innovate. While blame avoidance has attracted growing scholarly attention in public administration, limited empirical research has explored how grassroots civil servants’ subjective perceptions of blame risk influence their innovative behavior, and the underlying mechanisms remain under-examined.

**Methods:**

This study investigates the relationship between perceived blame risk and innovative behavior using survey data from 615 grassroots civil servants in China. It further examines the mediating role of career calling and the moderating role of perceived organizational support.

**Results:**

The results show that perceived blame risk is negatively associated with innovative behavior. Career calling partially mediates this relationship: higher perceived blame risk is associated with lower career calling, which, in turn, is associated with lower innovative behavior. Moreover, perceived organizational support moderated the negative associations between perceived blame risk and both career calling and innovative behavior, such that these associations were weaker at higher levels of perceived organizational support.

**Discussion:**

These findings highlight the importance of value-oriented career calling cultivation and supportive organizational safeguards in helping sustain grassroots civil servants’ innovative behavior under accountability pressure.

## 1 Introduction

Government innovation has become an essential strategy for strengthening governance capacity and navigating the profound changes unseen in a century (Hansen and Pihl-Thingvad, 2019). Grassroots civil servants constitute a key interface between the state and citizens. Their innovative behavior is therefore central to the functioning of the government innovation system. Prior research suggests that grassroots-level innovation can help bridge the gap between top-down reform initiatives and citizens’ actual needs (Lavee et al., 2018), while directly influencing policy implementation and the quality of government-citizen relations (Miao et al., 2018).

Despite the growing importance of innovation, from an individual civil servant’s perspective, this process may challenge established goals, routines, relationships, norms, and expectations within and beyond the administrative system (Bysted and Jespersen, 2014). Its process and outcomes are therefore difficult to predict. When innovative efforts fail to deliver expected results, the individuals involved may become exposed to blame. The risk of blame is a primary concern for grassroots civil servants. Once blame is attributed, civil servants may face reputational harm, hindered career progression, or other negative consequences (Weaver, 1986; Yao and Qian, 2025). Accordingly, scholars have often posited that civil servants are reluctant to innovate when they anticipate blame for failure (e.g., Lægreid et al., 2011; De Vries et al., 2016). Nevertheless, this assumption remains largely theoretical, with limited direct empirical evidence. More importantly, existing studies have predominantly examined organizational-level antecedents of innovative behavior, such as the organizational environment (Liu and Li, 2021), accountability arrangements (Tan and Jiang, 2025), and leadership styles (e.g., Yudiatmaja et al., 2023; Kousina and Voudouris, 2023). There is comparatively little understanding of how civil servants interpret these institutional arrangements and how such subjective perceptions shape their behavior. Therefore, this study shifts the analytical focus from objective accountability arrangements to subjective perceptions of blame risk and poses the following question: Is perceived blame risk associated with grassroots civil servants’ innovative behavior, and if so, how?

Furthermore, the mechanism underlying the relationship between perceived blame risk and innovative behavior also remains insufficiently understood. Innovation is both resource-intensive and resource-generating: it requires individuals to invest valuable resources (Zhu and Song, 2023), yet it can also create opportunities to acquire new resources (Liu et al., 2018). Drawing on conservation of resources (COR) theory, we argue that perceived blame risk may be negatively associated with innovative behavior by threatening and depleting individual resources. We focus specifically on career calling, an important individual psychological resource (e.g., Creed et al., 2014; Dalla Rosa et al., 2020; Vianello et al., 2020) reflecting the extent to which individuals perceive their work as meaningful, socially valuable, and conducive to self-realization. Career calling has been shown to be significantly associated with work engagement (Xie et al., 2017), innovative behavior (Zhou et al., 2020; Liu and Xu, 2022; Huang and Zhang, 2024), and various extra-role and proactive behaviors (Caillier, 2016). It may therefore serve as a proximal mechanism connecting perceived blame risk to grassroots civil servants’ innovative behavior. Accordingly, we ask: Is perceived blame risk associated with grassroots civil servants’ innovative behavior through career calling, and if so, how?

Perceived organizational support (POS) may further determine this relationship. POS refers to employees’ general belief that their organization values their contributions and cares about their well-being (Eisenberger et al., 1986). It serves as an important resource for alleviating work-related stress and promoting career development (Rhoades and Eisenberger, 2002), and can offset the negative effects of work demands. Employees with higher POS typically perceive greater access to external resources (Wen and Hou, 2018), develop stronger organizational identification, and invest more effort in maintaining or improving their work situation. We therefore expect POS to serve as a resource-buffering mechanism that mitigates psychological resource loss associated with high perceived blame risk. This consideration leads to the third research question: Does perceived organizational support moderate the negative associations involving perceived blame risk, and if so, how?

To address these questions, this study draws on survey data from 615 grassroots civil servants in China to systematically examine the relationship between perceived blame risk and innovative behavior. Specifically, we investigate whether career calling mediates this relationship and whether POS moderates the relevant pathways.

The paper is organized as follows. The following section reviews the relevant literature on perceived blame risk, innovative behavior, career calling, and perceived organizational support and develops the research hypotheses. Section 3 presents the data and the methodology used. Section 4 reports the empirical results, and Section 5 discusses the findings and outlines their theoretical and practical implications.

## 2 Literature and hypotheses

### 2.1 Perceived blame risk

Hood (2010:6) introduced the concept of “blame risk” in his work The Blame Game, defining it as the possibility of personal blame faced by public officials-including politicians, managers, professionals, and frontline staff-to distinguish it from terms such as reputational risk and political risk, which are typically associated with corporations or investors. However, the objective existence of such risks does not automatically translate into behavioral responses. Research on risk perception has shown that individuals’ assessments of risk are not based solely on rational calculations. Rather, they are determined by subjective interpretations, such that individuals may evaluate the same objective risk differently (Slovic, 1987). Accordingly, we further conceptualize blame risk as a subjective variable and adopt the term “perceived blame risk,” referring specifically to grassroots civil servants’ assessments of the possibility that they will be personally blamed for service failures, work-related errors, or other adverse outcomes.

Although perceived blame risk is conceptually related to felt accountability, fear of failure, job insecurity, and performance pressure, these constructs are substantively distinct. Distinguishing among them is important for clarifying the conceptual meaning and boundaries of perceived blame risk. First, felt accountability refers to an implicit or explicit expectation that one’s decisions or actions will be evaluated by salient audiences and that rewards or sanctions may follow from such evaluations (Hall and Ferris, 2011). Similar to perceived blame risk, felt accountability captures individuals’ subjective interpretations of objectively existing accountability arrangements. The identical formal accountability mechanisms may therefore elicit different perceptions among different individuals (Overman and Schillemans, 2022). However, felt accountability concerns the general expectation that one will be evaluated and may be required to justify one’s actions. Such evaluations may produce either favorable or unfavorable outcomes. Perceived blame risk is narrower in scope, as it focuses specifically on the prospect of being personally blamed for negative outcomes. Second, fear of failure refers to the negative emotions generated when an individual appraises failure as threatening, including guilt, shame, and embarrassment (Conroy, 2003). It is essentially an affective response to the anticipated consequences of failure, while perceived blame risk emphasizes the cognitive judgment regarding the possibility of personal blame. Third, job insecurity concerns perceived threats to the continuity and stability of one’s employment (Shoss, 2017). Its central focus is whether an individual’s job and career prospects remain secure. By contrast, perceived blame risk concerns the possibility of personal blame following an adverse work outcome and does not equal a threat of job loss. This distinction is particularly relevant in the Chinese civil service setting, where civil servants generally enjoy relatively high employment security, and dismissal is comparatively uncommon. Finally, performance pressure arises when employees perceive that their performance is tied to consequential rewards or sanctions and that they must meet demanding performance standards (Mitchell et al., 2018). Perceived blame risk has a wider set of potential triggers. It may arise not only from failure to meet performance targets, but also from procedural deviations, service failures, work-related errors, and other adverse outcomes that may attract blame. To summarize, perceived blame risk is not a general form of work stress or negative emotion. It is a cognitive construct embedded in public-sector accountability contexts and characterized by the anticipated attribution of personal responsibility for negative outcomes.

### 2.2 Perceived blame risk and innovative behavior among grassroots civil servants

Being blamed is generally experienced as undesirable and may threaten public officials’ career prospects. Public officials, therefore, have incentives to follow strategies that minimize their exposure to blame. Such strategies are commonly described as blame avoidance behavior (Weaver, 1986; Hood, 2010). Research on blame avoidance suggests that both the presence and intensity of blame risk affect public officials’ behavior (Hood, 2010). When officials perceive a higher risk of blame, they are more likely to adopt blame-avoidance strategies (e.g., Qin, 2022; Liu et al., 2025). Innovative behavior, however, stands in clear tension with blame avoidance. Innovation involves identifying problems, generating new ideas or solutions, mobilizing support, and implementing and disseminating those ideas (Scott and Bruce, 1994). By nature, this process is uncertain and risk-laden (Singh, 1986), making it difficult for actors to predict the outcomes and consequences of their actions in advance (Timeus and Breaugh, 2023). Furthermore, innovation projects are more likely to fail and to experience resource overruns (Tidd and Bessant, 2018). For grassroots civil servants, therefore, the perceived risk of blame may make innovation particularly unattractive. When perceived blame risk is high, their concern is not limited to whether an innovative initiative will fail, but extends to the possibility that failure will be followed by personal blame. To prevent being caught in a cycle of “innovation failure–blame attribution,” grassroots civil servants may prefer conservative, procedurally safe, and low-risk courses of action. As a result, they are less likely to engage in innovative attempts.

Conservation of resources (COR) theory further clarifies how perceived blame risk influences innovative behavior. This theory has been widely applied to explain individuals’ responses to environmental stressors and the coping behaviors they adopt (Westman et al., 2004). Its central premise is that individuals strive to acquire, maintain, foster and protect valuable resources (Hobfoll, 1989). Stress arises when resources are threatened with loss, actually lost, or invested without producing the expected returns (Hobfoll, 1989; Hobfoll et al., 2018). At the same time, individuals must invest resources to prevent further loss or to recover from prior losses (Hobfoll, 2001). However, the resource investment strategies available to individuals depend on their existing resource reserves. Compared to individuals with abundant initial resources, those with limited resources or already experiencing resource loss are more vulnerable to further losses. They are therefore more likely to prioritize protecting their remaining resources and become cautious about uncertain investments (Hobfoll, 2001; Hobfoll et al., 2018). Innovative behavior requires individuals to depart from path-dependent routines formed by prior experience. For grassroots civil servants, this entails not only investing substantial psychological resources and cognitive effort but also assuming the risks associated with outcomes that are difficult to predict accurately (Scott and Bruce, 1994). Furthermore, the negative consequences of perceived blame risk may place civil servants under considerable psychological strain, eliciting tension, anxiety, and frustration, thereby depleting their psychological resources. Such resource depletion has been associated with work withdrawal and short-term behavioral responses (Edmondson et al., 2019). Consequently, we posit that in contexts of high perceived blame risk, grassroots civil servants are more likely to adopt defensive resource-investment strategies to conserve their remaining resources (Hobfoll et al., 2018). They may therefore reduce the resources allocated to high-risk innovative activities, resulting in lower levels of innovative behavior. Taken together, we propose the following hypothesis:

*H1*: Perceived blame risk is negatively associated with grassroots civil servants’ innovative behavior.

### 2.3 Career calling

#### 2.3.1 Career calling as a resource

Career calling refers to a profound sense that one’s work is meaningful, socially valuable, and closely aligned with one’s identity (Dobrow and Tosti-Kharas, 2011). Due to its positive implications for proactive behavior, career success, and work-related well-being, this construct has received increasing attention in organizational behavior research (Zhou et al., 2020). Early studies often conceptualized career calling as a relatively stable, trait-like construct (Duffy et al., 2014) that develops gradually over time (e.g., Dobrow, 2013; Dobrow and Heller, 2015). Subsequent qualitative work, however, suggests that career calling may also fluctuate over shorter periods. In particular, crisis situations may strengthen individuals’ career calling when social needs become more salient, and their professional skills are perceived as critical for addressing those needs (Kent, 2019). Using longitudinal data, Vianello et al. (2020) further showed that career calling is not a fixed personal attribute rooted in stable dispositions or values; rather, it is a state shaped through ongoing interactions between individuals and their environments and can therefore be influenced by a range of factors (Dobrow, 2013). In recent years, this dynamic perspective has increasingly informed research on the antecedents of career calling. Existing literature has identified several antecedents, including individual gratitude traits (Li et al., 2023) and vocational identity (Dalla Rosa et al., 2019), as well as external factors such as social support (Dalla Rosa et al., 2019), person-organization fit, and person-job fit (Xu et al., 2023). Nevertheless, compared with the extensive literature on the consequences of career calling, research on its antecedents remains relatively limited. Even less is known about how career calling is formed and changes among civil servants.

The dynamic nature of career calling also provides a foundation for understanding it as a resource. Recent research suggests that career calling may take different forms, focus on different domains, and vary in duration across the life course (Zhou et al., 2024), indicating that it may be a malleable resource (Dalla Rosa et al., 2020; Vianello et al., 2020). This view has gained increasing empirical support. For example, drawing on the job demands–resources model, Creed et al. (2014) treated career calling as an indicator of personal resources and examined how it buffered the association between academic stress and academic burnout among junior doctors. Dalla Rosa et al. (2020) found that when psychological resources such as optimism, self-esteem, and perseverance were low, career calling served as an important personal resource that helped individuals maintain high-quality job-search behavior. Nielsen et al. (2020) further showed that career calling may function both as a buffering resource against work–family conflict and as a substitute resource when work–family enrichment is lacking.

Although the studies above conceptualize calling as a resource, they often do not clearly articulate the rationale for this perspective. According to the Conservation of Resources (COR) theory, individuals must invest resources to acquire new resources (Hobfoll et al., 2018). From this standpoint, Terry and Cigularov (2022) argued that a construct qualifies as a resource when it enables individuals to obtain other valued outcomes. Career calling can be considered a personal resource because it facilitates the development of self-efficacy, work competence, and work motivation. Mauno et al. (2025) further positioned career calling within the work stress process. Their meta-analysis, based on the job demands–resources model, demonstrated that career calling can function not only as an antecedent or moderator of work-related states, but also as a mediating mechanism linking work stressors to subsequent work outcomes (e.g., Mauno et al., 2022; Zhang et al., 2015; Zhou, 2024). In our study, perceived blame risk can be understood as a resource-threatening stressor. We therefore propose that career calling may serve as an important mechanism linking perceived blame risk to innovative behavior.

#### 2.3.2 The mediating role of career calling

Conceptually, career calling encompasses three core elements: (1) career calling is a transcendent calling or guiding force; (2) career calling is closely associated with the pursuit of meaning and self-actualization through work (Dobrow and Tosti-Kharas, 2011; Praskova et al., 2014); (3) career calling reflects an altruistic and prosocial orientation, whereby individuals seek to help others or contribute to the broader social good through their occupational roles (Duffy and Dik, 2013). Accordingly, grassroots civil servants are more likely to experience a strong sense of career calling when they view their work as meaningful, socially valuable, and conducive to the realization of personal and public values. However, career calling does not emerge in a vacuum. Rather, it is a dynamic construct that varies along a continuum from weaker to stronger levels (Dobrow and Tosti-Kharas, 2011; Creed et al., 2016; Dik et al., 2015; Dobrow, 2013). According to COR theory (Hobfoll, 1989), grassroots civil servants who perceive a high level of blame risk are more likely to interpret accountability as a threat to resource loss, which may trigger feelings of stress and tension. Under such circumstances, they are less likely to invest resources in exploring and developing their career calling. Moreover, a highly controlling institutional environment may undermine civil servants’ sense of self-determination and crowd out their intrinsic motivation (Frey and Jegen, 2001). As a result, they are likely to devote more attentional and psychological resources to risk monitoring, blame avoidance, and loss control, rather than to pursuing work-related meaning and self-actualization. In conclusion, this study proposes that:

*H2a*: Perceived blame risk is negatively associated with career calling.

Furthermore, as an important psychological resource, career calling can provide a sustained foundation for innovative behavior among grassroots civil servants. A substantial body of evidence has linked career calling to a range of positive outcomes, including career maturity, career adaptability, job and life satisfaction, and well-being (Thompson and Bunderson, 2019). For grassroots civil servants with a strong sense of career calling, work is not simply a means of fulfilling assigned duties. It also provides an avenue for realizing personal values, responding to public needs, and promoting social welfare. This sense of meaning and intrinsic responsibility can strengthen their psychological resilience in the face of difficulties and uncertainty, making them more willing to invest resources such as time, experience and cognitive effort in improving their work and adopting innovative practices. Simultaneously, this psychological resource can be further transformed into emotional resources, enhancing individuals’ job satisfaction and thereby fostering proactive and extra-role behaviors (Caillier, 2016; Esteves and Lopes, 2017). However, some studies have noted that employees with a strong sense of career calling may develop greater aspirations for professional growth and set more demanding standards for their work. Under such conditions, career calling may become an internal source of pressure, prompting employees to invest greater effort, strive for higher-quality task performance, and even exhibit workaholic tendencies (Keller et al., 2016). The resulting workload may, in turn, constrain their innovative behavior. However, this potential downside of career calling is more likely to emerge in highly competitive, performance-oriented organizational environments. By contrast, the public sector is generally characterized by relatively stable career trajectories and institutionalized performance appraisal systems, making it less likely that civil servants can achieve rapid career advancement solely through intensive work effort. Accordingly, in the public-sector context, career calling is more likely to function as a facilitating psychological resource than as a source of resource depletion. Based on the above reasoning, we propose that:

*H2b*: Career calling is positively associated with grassroots civil servants’ innovative behavior.

Combining H2a and H2b, this study further proposes a mediating hypothesis regarding career calling:

*H2*: Career calling mediates the relationship between perceived blame risk and grassroots civil servants’ innovative behavior.

### 2.4 The moderating role of perceived organizational support

Perceived organizational support (POS) refers to employees’ general belief that their organization values their contributions and cares about their well-being (Eisenberger et al., 1986). POS is commonly understood through the lens of social exchange: when employees perceive that their organization supports and values them, they are more likely to reciprocate through stronger commitment, greater effort, and constructive work behavior (Wayne et al., 1997). Consistent with this argument, previous studies indicate that employees with high POS are more willing to demonstrate creativity at work (Zhang et al., 2016), engage in proactive change behavior (Ma et al., 2023), and exhibit risk-taking behavior (Neves and Eisenberger, 2014).

From a COR-theory perspective, POS also constitutes a valuable social resource. It can, to some extent, compensate for the depletion of psychological resources caused by negative work experiences, such as the stress arising from perceived blame risk. By increasing individuals’ resource reserves, POS enhances their capacity to tolerate uncertainty and cope with potential losses, thereby encouraging more proactive resource-investment strategies (Marchand and Vandenberghe, 2014). At the same time, civil servants with high levels of POS are more likely to believe that their organization will evaluate their performance objectively and provide the necessary understanding and support when they encounter difficulties or make mistakes. This belief not only alleviates the stress associated with perceived blame risk and the resulting resource depletion (Ott et al., 2019) but also reduces the likelihood that individuals will interpret blame risk as a threat to their resources. Under these conditions, grassroots civil servants are more likely to view blame risk as a challenge that can be managed and overcome rather than as a threat that must be avoided. Accordingly, we propose the following hypothesis:

*H3*: POS attenuates the negative association between perceived blame risk and grassroots civil servants’ innovative behavior. Specifically, for grassroots civil servants with high POS, the negative association between perceived blame risk and innovative behavior is weaker (compared to those with low POS).

POS may also mitigate the negative association between perceived blame risk and career calling. First, from the conservation of resources perspective, the threat of resource loss associated with high perceived blame risk may prompt grassroots civil servants to allocate greater cognitive and emotional resources toward avoiding mistakes and preventing blame. Perceived organizational support, by contrast, provides additional emotional support, instrumental assistance, and resource replenishment. This support can expand individuals’ resource reserves and decrease their sensitivity to resource loss. Consequently, even under high perceived blame risk, grassroots civil servants with elevated levels of perceived organizational support may maintain the psychological resources needed to search for purpose, derive meaning from their work, and sustain their career calling.

Second, under conditions of high perceived blame risk, grassroots civil servants may interpret their relationship with the organization as adversarial, focused on blame attribution, rather than as a cooperative relationship oriented toward serving citizens and creating public value. This adversarial interpretation may diminish their sense of work meaningfulness, perceived value, and organizational fit. In such contexts, perceived organizational support signals that the organization values their contributions, prioritizes their well-being, and acknowledges the public value of their work (Eisenberger et al., 1986). When grassroots civil servants perceive this support, they are more likely to reciprocate with positive work-related attitudes and behaviors (Eisenberger et al., 1986), fostering an exchange relationship based on trust, commitment, and affective investment. This relationship can strengthen their organizational connection and identification, thereby alleviating the perceived misfit between the individual and the organization when the perceived blame risk is high. Prior research has demonstrated that such misfit is associated with lower levels of career calling (Xu et al., 2023). Based on this reasoning, the following hypothesis is proposed:

*H4*: POS attenuates the negative association between perceived blame risk and grassroots civil servants’ career calling. Specifically, for grassroots civil servants with high POS, the negative association between perceived blame risk and career calling is weaker (compared to those with low POS).

**Figure 1:**
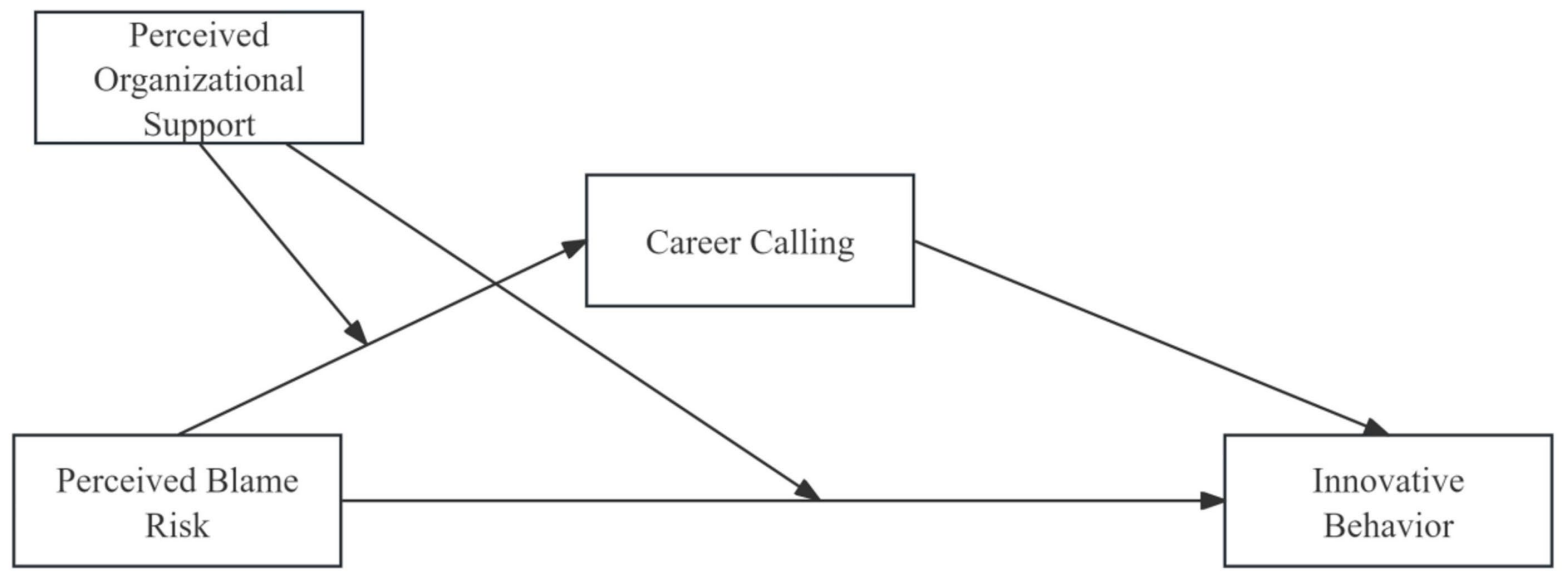
Theoretical model.

Figure 1 presents the theoretical model of this study.

## 3 Methods

This study combined convenience sampling with stratified random sampling. With the assistance of the Organization Department of the Municipal Committee in a major city in western China, the survey was administered during a training program for grassroots civil servants in one district. Based on China’s civil service position classification system, participants were stratified into three categories: administrative law enforcement positions, general administrative positions, and professional and technical positions. Respondents were then randomly sampled within each stratum. A total of 674 questionnaires were distributed. After excluding questionnaires containing missing or invalid responses, 615 valid responses were retained, yielding a usable-response rate of 91.24%. The final sample comprised 316 men (51.4%) and 299 women (48.6%). Of the respondents, 388 (63.1%) were younger than 35 years, whereas 227 (36.9%) were aged 35 years or older. 95 respondents (15.4%) had more than 20 years of work experience, and 520 (84.6%) had 20 years of work experience or less. Regarding educational attainment, 5 respondents (0.8%) had completed high school, secondary vocational education, or lower levels of education; 28 (4.6%) held associate degrees; 436 (70.9%) held bachelor’s degrees; and 146 (23.7%) held master’s degrees or higher qualifications. In terms of administrative rank, 276 respondents (44.9%) were at the staff-member rank or below, 231 (37.5%) were at the section-level rank, and 108 (17.6%) were at the division-level rank or above. Compared with the national distribution of civil servants, the sample was generally more highly educated and more senior in administrative rank. This difference may reflect the fact that the respondents were predominantly district-level civil servants participating in a training program. The findings should therefore be generalized to the broader population of Chinese civil servants with caution.

### 3.1 Materials

All constructs were measured using five-point Likert-type scales. Respondents rated each item from 1 to 5.

Independent variable: perceived blame risk. No validated scale is currently available for measuring perceived blame risk among grassroots civil servants in the Chinese context. We therefore adapted the measure developed by Klijn et al. (2022). Consistent with their conceptualization, perceived blame risk captures the extent to which civil servants believe that they may be personally blamed for problems arising from their work. The scale assesses blame risk from three potential sources: supervisors, colleagues, and the public. It consists of three items: “I run the risk that problems will be blamed on me personally by my supervisor / colleagues / the public” (see Appendix). The scale was adapted using a standard translation and back-translation procedure. First, two researchers proficient in English independently translated the original scale into Chinese. Their translations were reviewed and reconciled to produce a preliminary Chinese version. Next, two additional researchers who had not been exposed to the original scale independently translated the Chinese version back into English. The research team compared the Chinese and back-translated versions and identified no ambiguous items. Two public administration scholars were then invited to assess the items’ content relevance, semantic accuracy, and contextual appropriateness. The scale was further revised in response to their feedback. Finally, a pilot test was conducted with several public administration students to assess the items’ comprehensibility and clarity, resulting in the final version used in the survey.

The Perceived Blame Risk Index was calculated as the mean of the three items, with higher scores indicating greater perceived blame risk. The scale demonstrated satisfactory internal consistency in the present study (Cronbach’s *α* = 0.886). An exploratory factor analysis extracted a single factor with an eigenvalue greater than 1, accounting for 81.664% of the total variance. These results support the unidimensionality of the scale and indicate that the three items reflect a common underlying construct.

Mediating variable: career calling. Career calling was measured using a translated version of the 12-item unidimensional Career Calling Scale (CQ12) developed by Dobrow and Tosti-Kharas (2011). Sample items included “I have a clear idea of what I want to accomplish in my work”, “I feel that my work is meaningful and serves a greater purpose.” The complete list of items is provided in the Appendix. The Career Calling Index was calculated as the mean of the 12 items, with higher scores indicating a stronger sense of career calling. The scale demonstrated satisfactory internal consistency in the present study (Cronbach’s α = 0.946).

Moderating variable: perceived organizational support. Perceived organizational support (POS) was measured using a six-item scale adapted from Eisenberger et al. (2001). A sample item is “My organization values my contribution to its well-being.” The complete list of items is provided in Appendix. The POS Index was calculated as the mean of the six items, with higher scores indicating greater POS. The scale demonstrated satisfactory internal consistency in the present study (Cronbach’s α = 0.892).

*Dependent variable*: innovative behavior. Innovative behavior was measured using the six-item scale developed by Scott and Bruce (1994). A sample item is “Searches out new technologies, processes, techniques, and/or product ideas.” The complete list of items is provided in the Appendix. The Innovative Behavior Index was calculated as the mean of the six items, with higher scores indicating higher levels of innovative behavior. The scale demonstrated satisfactory internal consistency in the present study (Cronbach’s α = 0.903).

### 3.2 Reliability and validity assessment

SPSS and AMOS were used to assess the reliability and validity of the four scales. The results are reported in Tables 1, 2. Cronbach’s α exceeded 0.85 for all constructs, indicating satisfactory internal consistency. The standardized factor loadings ranged from 0.566 to 0.891. The minimum composite reliability (CR) value exceeded 0.80, and the minimum average variance extracted (AVE) value exceeded 0.60, indicating adequate convergent validity. In addition, the square root of the AVE for each construct was greater than its correlations with the other constructs, providing evidence of discriminant validity.

**Table 1:** Reliability and convergent validity results.

Variables	Cronbach’s *α*	Standardized factor loadings	CR	AVE
Perceived Blame Risk	0.886	0.829–0.875	0.888	0.725
Career Calling	0.946	0.655–0.874	0.948	0.605
Perceived Organizational Support	0.892	0.566–0.891	0.900	0.605
Innovative Behavior	0.903	0.724–0.850	0.904	0.611

**Table 2.** Correlation matrix and square roots of AVE.

Variables	Perceived blame risk	Career calling	Perceived organizational support	Innovative behavior
Perceived blame risk	**0.851**			
Career calling	-0.071^*^	**0.778**		
Perceived organizational support	-0.008	0.325^***^	**0.778**	
Innovative behavior	-0.130^***^	0.288^***^	0.114^***^	**0.782**

Note: Bold values on the diagonal are the square root of the AVE. ^***^and ^*^indicate significance at the 0.001 and 0.05 levels, respectively.

A confirmatory factor analysis (CFA) conducted in AMOS showed that the hypothesized four-factor measurement model comprising 27 items showed: χ^2^ = 1240.201, df = 318, χ^2^/df = 3.900, RMSEA = 0.069, CFI = 0.921, TLI = 0.913, NFI = 0.897, and IFI = 0.921. Although the NFI was slightly below the conventional threshold of 0.90, it was very close to this value, and the other fit indices met commonly accepted criteria. Overall, the measurement model demonstrated an acceptable fit to the data.

## 4 Results

Several procedural and statistical approaches were used to assess and mitigate potential common method bias. At the data-collection stage, the survey was administered anonymously, and selected items were reverse-coded. Harman’s single-factor test was then conducted using an unrotated exploratory factor analysis of all measurement items. Four factors with eigenvalues greater than 1 were extracted. The largest factor accounted for 38.048% of the total variance, suggesting that no single factor dominated the covariance among the items.

An unmeasured latent methods factor (ULMC) test was also conducted, using the four-factor measurement model as the baseline. Adding a latent method factor did not materially improve model fit: χ^2^ = 1240.079, df = 317, χ^2^/df = 3.912, RMSEA = 0.069, CFI = 0.921, TLI = 0.906, NFI = 0.897, and IFI = 0.921. Taken together, these results indicate that common method bias is unlikely to pose a serious threat to the validity of the findings.

Before conducting the regression analyses, we assessed whether the data satisfied the relevant model assumptions. First, the normality of the residuals was examined using a normal probability plot of the standardized residuals. The residuals approximately followed a normal distribution. And given the relatively large sample size (*N* = 615), minor deviations from normality were unlikely to materially affect the statistical significance of the regression estimates. Second, homoscedasticity was assessed using a scatterplot of standardized residuals against standardized predicted values. The residuals were randomly distributed around zero and displayed no clear funnel-shaped pattern of expansion or contraction, indicating no substantial heteroscedasticity. Third, the independence of the error terms was assessed using the Durbin–Watson statistic. The value was 1.879, close to the theoretical benchmark of 2, indicating no substantial autocorrelation in the residuals. Finally, the variance inflation factor (VIF) values for all predictors were no greater than 1.4, indicating no serious multicollinearity.

Overall, the data satisfied the assumptions of residual normality, homoscedasticity, independence of errors, and the absence of serious multicollinearity. The data were therefore suitable for subsequent regression analyses.

### 4.1 Descriptive statistics and correlation analysis

Table 3 presents the descriptive statistics for the main study variables. The correlation analysis showed that perceived blame risk was negatively correlated with innovative behavior (*r* = −0.231, *p* < 0.01) and career calling (*r* = −0.090, *p* < 0.05). Career calling was positively correlated with POS (*r* = 0.462, *p* < 0.01) and innovative behavior (*r* = 0.456, *p* < 0.01). POS was also positively correlated with innovative behavior (*r* = 0.253, *p* < 0.01). Overall, the correlations were consistent with the hypothesized relationships.

**Table 3:** Descriptive statistics.

Variables	M	SD
Perceived blame risk	3.312	0.952
Career calling	3.692	0.798
POS	3.612	0.796
Innovative behavior	3.940	0.715

### 4.2 The relationship between perceived blame risk and innovative behavior: the mediating role of career calling and the moderating role of POS

We first used Model 4 of the PROCESS macro for SPSS (Hayes, 2017) to examine the indirect association between perceived blame risk and innovative behavior through career calling, controlling for gender, age, working years, education, and position. As shown in Table 4, perceived blame risk was negatively associated with innovative behavior (Model 3: b = −0.172, t = −5.845, *p* < 0.001), supporting H1. Perceived blame risk was also negatively associated with career calling (Model 1: b = −0.071, t = −2.127, *p* < 0.05), supporting H2a. In turn, career calling was positively associated with innovative behavior (Model 2: b = 0.387, t = 11.929, *p* < 0.001), supporting H2b. The bootstrap confidence intervals for both the direct association between perceived blame risk and innovative behavior and the indirect association through career calling excluded zero. The direct association was −0.145, accounting for 84.30% of the total association. The indirect association through career calling was −0.027, accounting for the remaining 15.70% of the total association (−0.172). These results suggest that career calling explained a meaningful proportion of the relationship between perceived blame risk and innovative behavior, which supports H2.

**Table 4:** Results of hypothesis testing.

Variable	Career calling (model 1)		Innovative behavior (model 2)		Innovative behavior (model 3)		Innovative behavior (model 4)		Career calling (model 5)	
	b	t	b	t	b	t	b	t	b	t
Gender	−0.171^**^	−2.614	0.002	0.040	−0.064	−1.103	−0.028	−0.496	−0.093	−1.595
Age	0.001	−0.095	0.016	0.348	0.017	0.317	0.000	0.005	−0.034	−0.644
Working years	−0.001	−0.123	−0.002	−0.170	−0.002	−0.207	0.001	0.122	0.005	0.507
Education	0.175^**^	2.895	0.045	0.920	0.113	2.088	0.098	1.885	0.151^**^	2.799
Post	−0.149^***^	−3.314	−0.016	−0.434	−0.073	−1.839	−0.049	−1.277	−0.106^*^	−2.662
Perceived blame risk	−0.071^*^	−2.127	−0.145 ***	−5.439	−0.172 ***	−5.845	−0.166 ***	−5.846	−0.058	−1.959
Career calling			0.387 ***	11.929						
POS							0.213***	6.228	0.447 ***	12.613
Perceived blame risk × POS							0.122**	3.301	0.114 **	2.981
Constant	3.811^***^	13.345	2.844***	10.944	4.319***	17.025	3.744***	16.636	3.561	15.270
*R^2^*	0.053		0.246		0.070		0.140		0.258	
*F*	5.675		28.344		7.588		12.296		26.323	

b represents unstandardized regression coefficients. ^***^, ^**^, and ^*^indicate significance at the 0.001, 0.01, and 0.05 levels, respectively.

Model 1 of the PROCESS macro for SPSS (Hayes, 2017) was used to examine whether POS moderated the association between perceived blame risk and innovative behavior. As shown in Model 4, the interaction term between perceived blame risk and POS was statistically significant and positive (*b* = 0.122, t = 3.301, *p* < 0.01). Given that the association between perceived blame risk and innovative behavior was negative, the positive interaction coefficient indicates that POS attenuated this negative association. These findings support H3.

Finally, we used Model 1 of the PROCESS macro for SPSS (Hayes, 2017) to examine whether POS moderated the association between perceived blame risk and career calling, with gender, age, tenure, educational attainment, and administrative rank included as control variables. As reported in Table 4, the interaction term between perceived blame risk and POS was positively associated with career calling (Model 5: b = 0.114, t = 2.981, *p* < 0.01). This positive interaction coefficient indicates that POS weakened the negative associations between perceived blame risk and career calling, which supports H4.

**Figure 2:**
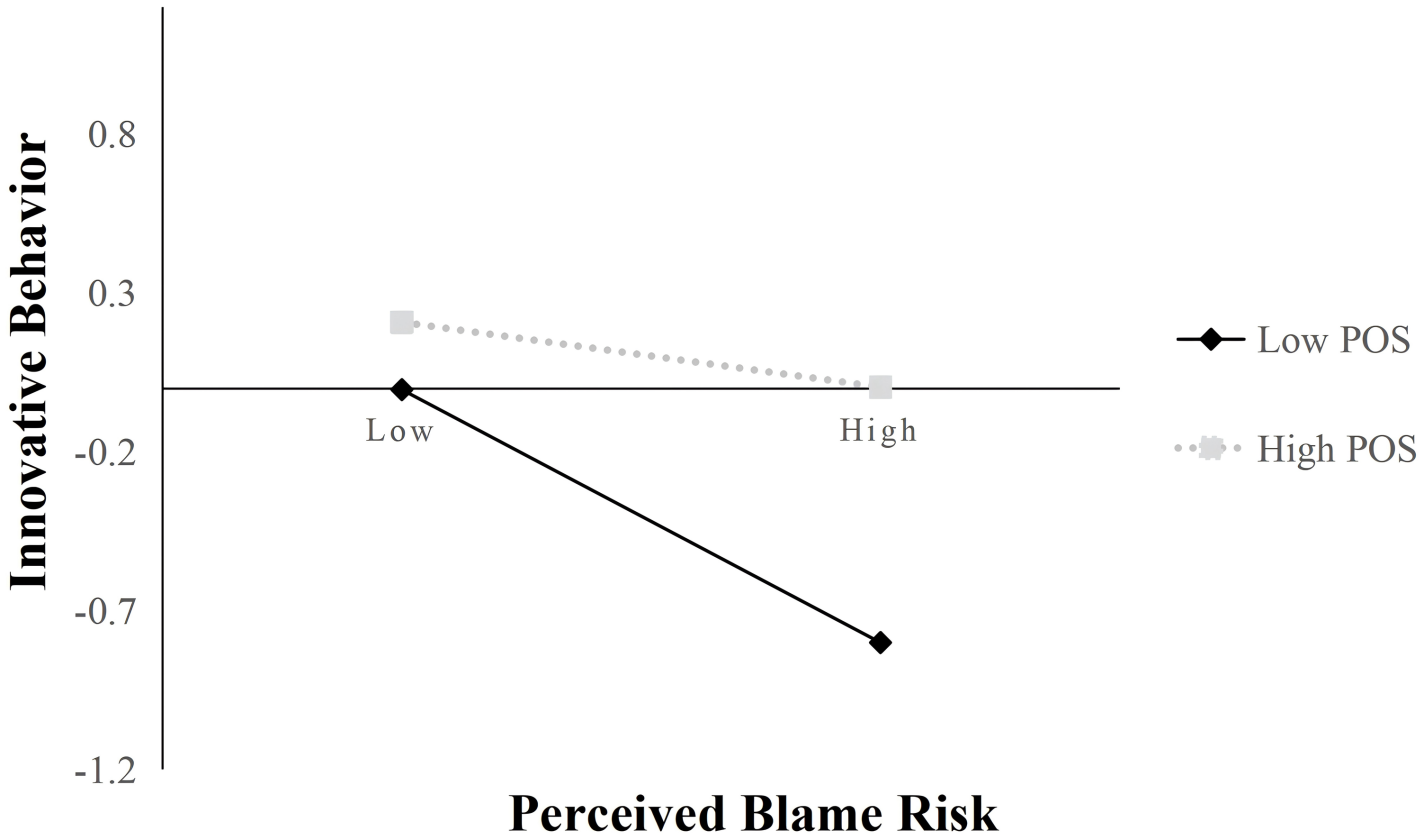
The moderating role of POS (mean-centered innovative behavior).

The simple-slope analysis provides further evidence for these patterns. As illustrated in Figure 2, when POS was low (M − 1 SD), perceived blame risk was significantly and negatively associated with innovative behavior. When POS was high (M + 1 SD), the association remained negative but was weaker. Thus, higher levels of POS attenuated the negative association between perceived blame risk and innovative behavior.

**Figure 3:**
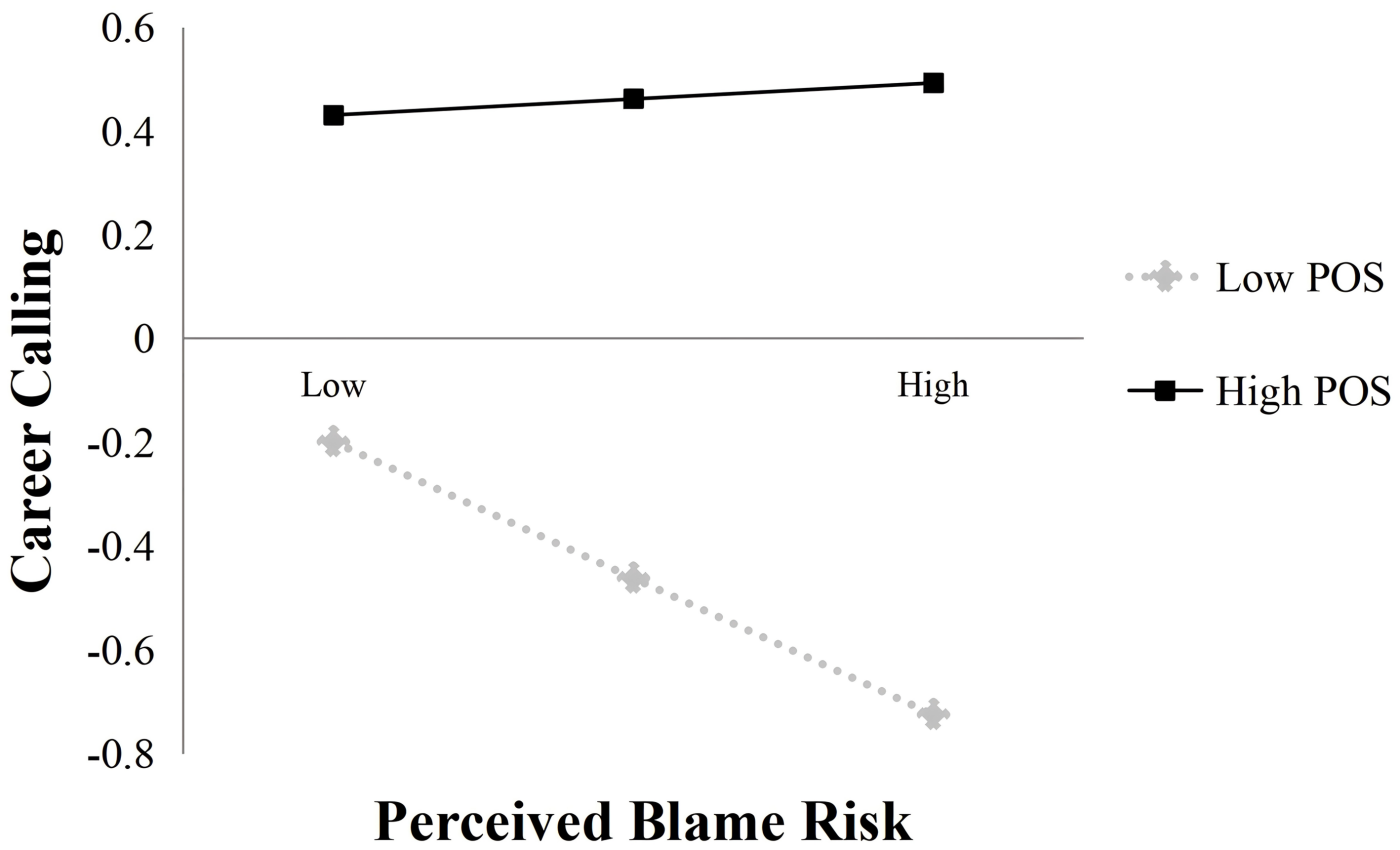
The moderating role of POS (mean-centered career calling).

As shown in Figure 3, when POS was low (M − 1 SD), perceived blame risk was significantly and negatively associated with career calling. When POS was high (M + 1 SD), the slope shifted upward. Taken together, the results indicate that higher levels of POS weakened the negative association between perceived blame risk and career calling.

## 5 Discussion

Drawing on questionnaire survey data, this study examined the relationship between grassroots civil servants’ perceived blame risk and innovative behavior, as well as the mediating and moderating patterns associated with this relationship. The findings show that perceived blame risk is negatively associated with innovative behavior. Career calling mediates this relationship. Perceived organizational support (POS) is associated with a weaker negative relationship between perceived blame risk and both innovative behavior and career calling. Although some of the observed associations were modest in magnitude, they are practically meaningful in the context of public employee innovation, which is determined by multiple individual, organizational, and institutional factors.

First, perceived blame risk is negatively associated with innovative behavior among grassroots civil servants. Specifically, when grassroots civil servants perceive that they are more likely to be blamed by supervisors, colleagues, or citizens for service failures, poor performance, or other adverse outcomes, they are less likely to engage in innovative behavior. Previous studies have shown that although accountability can enhance transparency and responsiveness in bureaucratic behavior, it can also generate unintended consequences, including inaction and inappropriate action intended to avoid blame (Tu, 2024). By shifting the analytical focus from objective accountability arrangements to civil servants’ subjective perceptions of blame risk, this study presents empirical evidence that perceived blame risk corresponds to a stronger tendency to avoid innovative action. This finding also complements recent research showing that institutional pressure can increase civil servants’ blame avoidance behavior by heightening their perceptions of risk (Liu et al., 2025). One plausible explanation is that innovative initiatives are inherently uncertain: they may fail to achieve their intended outcomes, and their actual results may deviate from initial expectations (Tidd and Bessant, 2018). In an accountability context, innovation involves not only experimenting with new working methods but also assuming the potential resource losses associated with trial and error (De Vries et al., 2016). Given their generally risk-averse tendencies, civil servants who perceive a high level of blame risk are more likely to focus on the possible consequences of failure, prioritize preserving existing resources, and avoid actions that may expose them to blame. In addition, the current institutional context in China may also provide grassroots civil servants with practical room to refrain from innovation. As early as 2003, innovative capacity was incorporated into China’s general competency framework for civil servants, and subsequent policy documents have repeatedly emphasized the need to strengthen civil servants’ awareness of innovation and encourage innovative behavior. Nevertheless, in practice, the civil-service appraisal system continues to rely primarily on the five dimensions of ‘morality, competence, diligence, performance and integrity’. Innovative performance is generally subsumed under the more general category of performance rather than assessed as a distinct criterion. Innovative behavior has therefore not been fully institutionalized as an expected in-role behavior among civil servants, but rather an extra-role or proactive behavior undertaken at the individual’s discretion (Miao et al., 2018). Consequently, grassroots civil servants retain considerable discretion over whether to engage in innovative practices. When high perceived blame risk suppresses their willingness to innovate, this attitudinal shift can more readily translate into behavioral choices that favor maintaining the status quo and avoiding experimentation.

Second, career calling mediates the relationship between perceived blame risk and innovative behavior. Specifically, higher levels of perceived blame risk are associated with lower levels of career calling, whereas career calling is positively associated with innovative behavior. These findings suggest that career calling is an important psychological mechanism linking perceived blame risk and innovative behavior. Although career calling has received sustained attention in organizational behavior research, it remains comparatively underexplored in public administration scholarship. Thompson and Christensen (2018) were among the first to systematically introduce career calling into public administration research and to compare it with public service motivation. They argued that career calling and public service motivation overlap conceptually and are complementary: both describe work orientations and motivations that go beyond narrow self-interest and prioritize others’ needs. However, career calling is not synonymous with public service motivation. Public service motivation is typically conceptualized as a relatively stable value orientation centered on serving the public interest and helping others (Perry and Wise, 1990; Perry, 1996; Vandenabeele, 2007), whereas career calling additionally emphasizes the pursuit of meaning, identity alignment, and self-realization through work, and it may be more sensitive to contextual influences. The empirical findings of this study further indicate that high perceived blame risk may direct civil servants’ attention toward potential failures and resource losses, thereby undermining their exploration and experience of occupational meaning and mission. This interpretation is consistent with the view that career calling is dynamic and develops through ongoing interactions between individuals and their environments (Vianello et al., 2020). It also provides new insights from the public-sector context to the still-limited literature on the antecedents of career calling. At the same time, the positive association between career calling and innovative behavior is consistent with previous research (Liu and Xu, 2022; Zhou et al., 2020). When grassroots civil servants derive a stronger sense of meaning, mission and social value from their work, they are more likely to engage in proactive behavior and accept the uncertainty inherent in innovation.

Finally, this study shows that POS plays an important moderating role in the relationship between perceived blame risk and innovative behavior. Previous research has documented a positive relationship between POS and employees’ innovative behavior (e.g., Zhou and Li, 2018; Zhang et al., 2016). When employees perceive that their organization values their contributions and cares about their well-being, they are more likely to adopt enthusiastic, proactive work attitudes and to reciprocate with constructive work behavior (Eisenberger et al., 1986; Rhoades and Eisenberger, 2002; Kurtessis et al., 2017). Moreover, POS also moderated the negative association between perceived blame risk and career calling. These patterns further suggest a conditional indirect association, whereby the negative indirect relationship between perceived blame risk and innovative behavior through career calling is weaker when POS is higher. Notably, when POS is high, the association between perceived blame risk and career calling shifts from negative to positive. Because this relationship does not reach statistical significance, it should be interpreted with caution. Nevertheless, the change in the slope’s direction offers a potentially informative clue. Recent accountability scholarship has increasingly focused on felt accountability, emphasizing that public officials do not passively respond to formal accountability arrangements. Rather, they interpret accountability signals through their own cognitive frames, construct meaning from those signals, and adjust their behavioral strategies correspondingly (Hall and Ferris, 2011; Aleksovska, 2021; Han and Perry, 2020). Drawing on the psychological mechanisms of motivation, Xiong and Xie (2025) distinguish between punitive and duty-promoting forms of accountability. The former emphasizes assigning responsibility and imposing severe sanctions, seeking to deter undesirable behavior by activating fear and risk aversion. The latter rely more heavily on positive incentives and aim to promote proactive learning and effective performance through learning by observation, capacity building, and environmental support. Consequently, we infer that high POS may activate a duty-promoting process, enabling grassroots civil servants to interpret accountability not simply as punitive control or a threat, but as an opportunity to clarify responsibilities, strengthen capabilities and improve work performance. This may foster a stronger sense of responsibility and value alignment, reinforce career calling, and encourage proactive behavior.

### 5.1 Theoretical implications

This study advances the literature on civil servants’ innovative behavior in two key ways. First, while previous research has primarily focused on objective organizational conditions such as formal accountability arrangements, we adopt an actor-centered perspective by examining civil servants’ subjective perceptions of blame risk. We further introduce career calling, a psychological resource that has received limited attention in public administration research, and identify its mediating role. This goes beyond a purely risk-aversion explanation for why accountability may inhibit innovation. Perceived blame risk may not only make civil servants more reluctant to assume the risks associated with innovation. It may also erode the sense of occupational meaning and value that motivates innovation. Thus, the challenge is not only that civil servants may not dare to innovate, but also that they may lack the intrinsic motivation to do so.

Second, this study discovers a significant boundary condition in the relationship between perceived blame risk and innovative behavior. Our results show that POS attenuates the negative association between perceived blame risk and innovative behavior among grassroots civil servants, suggesting that accountability does not uniformly inhibit innovation. A supportive organizational environment may help reduce the perceived threat of blame and enable civil servants to interpret accountability as a manageable challenge and an opportunity for learning and improvement, rather than solely as a mechanism of control and punishment. This insight offers a more nuanced theoretical perspective for reconciling accountability with innovation in the public sector.

### 5.2 Practical implications

The above findings offer several implications for public administration practice. First, strengthening accountability should not be equated with increasing the severity of sanctions. Accountability systems should strike an appropriate balance between holding civil servants responsible and protecting those who act in good faith. Public organizations should develop duty-promoting accountability arrangements and foster an accountability mindset centered on commitment, thereby encouraging civil servants to take initiative. While civil servants should be to blame for inaction, inappropriate action, and clear dereliction of duty, accountability standards should be clear, stable, and predictable. A single outcome should not serve as the sole basis for determining whether an individual has fulfilled their responsibilities. This is particularly important for innovative tasks characterized by substantial uncertainty. In such cases, assessments should take into account civil servants’ motives, the processes through which they performed their duties, the objective conditions they faced, and the effort they invested. It is essential to distinguish violations and dereliction of duty from unintentional errors arising from good-faith experimentation. Second, the positive role of POS suggests that developing a supportive organizational atmosphere is an important means of encouraging innovative behavior among civil servants. Public organizations should provide grassroots civil servants with adequate work-related support and safeguard their legitimate rights and interests. So, even in accountability contexts, civil servants can recognize the meaning and value of their work, sustain a strong sense of career calling, actively fulfill their responsibilities, and take initiative.

## 6 Limitations and directions for future research

This study has several limitations that point to promising avenues for future research. First, accountability mechanisms can be differentiated along two dimensions: accountability form and accountability focus. Specifically, they may be reward- or punishment-oriented and process- or outcome-oriented (Zhang et al., 2026). This study focuses only on the blame that grassroots civil servants may face for service failures, poor performance, work-related errors, and other adverse outcomes. It therefore concentrates on a form of accountability that is primarily punishment- and outcome-oriented. Given the differences in the logic of behavioral motivation and constraint across accountability mechanisms, future research should explore perceived blame risk and its behavioral consequences under reward- and process-oriented accountability. Second, this study considers only the moderating role of POS. Future research could incorporate the moderating effects of personal characteristics, such as personality traits, risk preferences, and risk tolerance, as well as organizational contextual factors, such as leadership styles and tolerance-for-error atmosphere, which would support a more comprehensive analytical framework and deepen our understanding of how grassroots civil servants’ innovative behavior develops under accountability arrangements. Third, this study employs a cross-sectional questionnaire survey design, in which all core variables are derived from individual self-reports collected at a single point in time. Although we used anonymous questionnaires, Harman’s single-factor test, and an unmeasured latent method factor test to evaluate and reduce common method bias, the potential influence of this bias cannot be entirely ruled out. Future research could employ longitudinal tracking designs, experimental methods, or multi-wave data collection to assess the robustness of the present findings more rigorously. Fourth, innovative behavior is measured mainly through respondents’ self-assessments. Because innovative behavior is socially desirable, respondents may overestimate their own level of innovation, resulting in social desirability bias. Future studies could incorporate multisource data, including supervisor evaluations, peer assessments, and objective performance indicators, to provide a more comprehensive assessment of innovative behavior and improve the accuracy and credibility of the findings. Finally, this study is situated in the Chinese public-sector context. China’s distinctive administrative system, accountability arrangements, and organizational culture may shape how grassroots civil servants perceive the risk of blame and respond behaviorally. The generalizability of the present findings should therefore be examined in other administrative and cultural settings.

## Data Availability

The raw data supporting the conclusions of this article will be made available by the authors, without undue reservation.
