# Supplementary material for: Perceived blame risk and innovative behavior among Chinese grassroots civil servants: the roles of career calling and perceived organizational support
Source: Front Psychol. 2026 Aug 5;17:1867276. doi: 10.3389/fpsyg.2026.1867276 (PMC13485813; doi:10.3389/fpsyg.2026.1867276)
Supplement: Supplementary file 1 [file Supplementary_file_1.docx]

# Appendix

Questionnaire

Dear leader:

Hello! Thank you for taking the time out of your busy schedule to fill out the questionnaire! This is an anonymous academic survey aimed at understanding the current work and related situation of government officials. All information is for overall statistical use only and will not have any adverse effects on you. Please answer with peace of mind! Your answer has a positive significance in improving the efficiency of government work. Thank you again for your support and assistance!

Graduate Research Project Team of School of Public Administration, Chongqing University

Part A: Please fill in the following information based on your personal reality. (The following demography variables are only used for statistical classification)

A-1. Your gender:	□ Male	□ Female

A-2. Your age:	□ 30 years old and below	□ 31-40 years old

□ 41-50 years old	□ 51 years old and above

A-3.Your working years: years.

A-4.Your educational background: □ High school, secondary vocational school, or lower

□ Associate degree	□ Bachelor's degree	□ Master's degree or above

A-5. Your position:	□ Division (Director/Deputy Director) Level and Above

□ Section (Chief/Deputy Chief) Level	□ Staff Level and Below

Part B：Please rate the following statements based on your true feelings in actual work, with a score of 1-5 representing your level of agreement. (1=completely disagree; 2=relatively disagree; 3=uncertain; 4=quite agree; 5=fully agree)

**Perceived Blame Risk Scale**

B-1. I run the risk that problems will be blamed on me personally by my superior.

B-2. I run the risk that problems will be blamed on me personally by my colleagues.

B-3. I run the risk that problems will be blamed on me personally by the public.

**Innovative Behavior Scale**

Please rate your performance in the following areas:

C-1. Searches out new technologies, processes, techniques, and/or product ideas.

C-2. Generates creative ideas.

C-3. Promotes and champions ideas to others.

C-4. Investigates and secures funds needed to implement new ideas.

C-5. Develops adequate plans and schedules for the implementation of new ideas.

C-6. Is innovative

**Career Calling Scale**

D-1. I have a clear idea of what I want to accomplish in my work.

D-2. I feel that my work is meaningful and serves a greater purpose.

D-3. My work reflects who I am and what I value.

D-4. My work is a good fit with my talents and abilities.

D-5. My work allows me to express myself creatively.

D-6. My work provides a sense of accomplishment and fulfillment.

D-7. I feel a sense of pride in my work.

D-8. My work feels like an important part of my life.

D-9. I am passionate about my work.

D-10. My work provides a sense of belonging and community.

D-11. My work feels like a calling.

D-12. I feel a sense of joy and enthusiasm when I think about my work.

**Perceived Organizational Support Scale**

E-1. My organization values my contribution to its well-being.

E-2. My organization really cares about my well-being.

E-3. My organization strongly considers my goals and values.

E-4. My organization really cares about my opinions.

E-5. My organization rarely pays attention to me.

E-6. My organization would help me if I had a work-related problem.
